# Evaluation of the Effect of Chitosan-Based Irrigation Solutions on the Bond Strength of Mineral Trioxide Aggregate to Bulk-Fill Composite

**DOI:** 10.3390/jfb15120370

**Published:** 2024-12-08

**Authors:** Arzu Şahin Mantı, Bağdagül Helvacıoğlu Kıvanç

**Affiliations:** Department of Endodontics, Faculty of Dentistry, Gazi University, 06490 Ankara, Türkiye; bagdagul@gazi.edu.tr

**Keywords:** chitosan, ethylenediaminetetraacetic acid, nanoparticles, cholorhexidine, mineral trioxide aggregate, bulk-fill composite, shear bond strength

## Abstract

**(1) Background:** Bond strength between repair and restorative materials is crucial for endodontic success. This study assessed the effects of the following final irrigation solutions on the bond strength of mineral trioxide aggregate (MTA) to a bulk-fill composite: (1) 17% Ethylenediamine tetraacetic acid (EDTA); (2) 2% Chlorhexidine (CHX); (3) 0.2% chitosan; (4) 0.2% chitosan with 2% CHX; 5) 0.2% chitosan with AgNPs. **(2) Methods**: Sixty MTA samples were divided into six groups (*n* = 10) based on the final irrigation solution: 1. EDTA, 2. CHX, 3. Chitosan, 4. Chitosan-CHX, 5. Chitosan-AgNP, and 6. distilled water (control). After a 5-min solution exposure, each sample was restored with the bulk-fill composite, and the shear bond strength (SBS) was measured. Structural changes in MTA were analyzed using SEM and EDS, and failure modes were classified as adhesive, cohesive, or mixed. Data were analyzed by one-way ANOVA with Tamhane’s T2 and Tukey’s tests (α = 0.05). **(3) Results:** EDTA exhibited the lowest SBS (*p* < 0.001), while Chitosan-CHX showed the highest. SEM showed a spongy, void-rich surface in EDTA-treated MTA, with significant Ca depletion per EDS. Chitosan-CHX showed no structural change. Cohesive fractures within MTA were predominant. **(4) Conclusions**: EDTA significantly reduces SBS, while chitosan with CHX enhances bond strength.

## 1. Introduction

Tricalcium silicate-based cements are favored in endodontic treatments for applications like perforation repair, resorption repair, apical plugs, and apexification, owing to their biocompatibility, capacity to promote hard tissue formation, and superior sealing properties [1]. Perforation repair is particularly crucial, as perforations account for approximately 10% of endodontic treatment failures [2]. These perforations can arise from iatrogenic causes or pathological conditions like root resorption [3]. Advances in materials and techniques for perforation management aim to enhance periodontal tissue healing and minimize complications [4]. Among these materials, mineral trioxide aggregate (MTA) stands out as one of the most effective tricalcium silicate cements [5]. The first tricalcium silicate-based cement introduced, ProRoot MTA (Dentsply, Tulsa Dental Products, OK, USA), is composed of tricalcium silicate, dicalcium silicate, tricalcium aluminate, tetracalcium aluminoferrite, gypsum, free calcium oxide, and bismuth oxide. However, it has been reported to have certain disadvantages, including prolonged setting time, potential tooth discoloration, difficult handling properties, and high cost. To address these limitations, alternative formulations such as Ortho-MTA (BioMTA, Seoul, Republic of Korea) have been developed. Ortho-MTA has a similar composition to ProRoot MTA, but has been reported to contain lower levels of heavy metals compared to its predecessor [6]. During chemomechanical preparation in endodontic procedures, fully set MTA is often exposed to various irrigation solutions, which can potentially alter its physical and chemical properties.

Ethylenediamine tetraacetic acid (EDTA) is widely used as a final irrigant in endodontics due to its ability to remove the smear layer and enhance bond strength in root canal fillings. However, it can negatively affect tricalcium silicate cements [7,8]. Chlorhexidine (CHX), another preferred irrigant, provides broad-spectrum antimicrobial effects, improves bond strength by inhibiting collagen-degrading enzymes, and exhibits high biocompatibility [9,10]. Chitosan, a natural polymer derived from marine sources, is emerging as an alternative irrigant. It effectively chelates and removes the smear layer with minimal dentin demineralization and has antimicrobial properties comparable to NaOCl and CHX [11,12]. Studies suggest that combining chitosan with CHX enhances antimicrobial efficacy and bond strength, making it a promising final irrigant option [11,12].

Silver nanoparticles (AgNPs) have recently gained attention for their biocompatibility and potent, long-lasting antimicrobial effects. When combined with chitosan, AgNPs create a chelating solution with synergistic antibacterial properties, which may improve endodontic outcomes [13,14].

Restoration following root canal treatment is critical for treatment success [15]. The technical quality of coronal restorations is more important in maintaining periapical health than the quality of the endodontic procedure itself [16]. This emphasizes the role of coronal restorations in preventing bacterial penetration, endotoxin, and salivary invasion [17]. Factors such as material loss in the tooth, choice of restorative materials, adhesive systems, irrigation solutions, temporary fillings, and canal pastes affect the quality of coronal restorations [15,16]. While layering techniques in composite resin restorations may cause failure due to interlayer issues, contamination, and voids [18,19], bulk-fill composites are increasingly favored [20,21]. These materials offer homogeneous distribution, reduced application time, and superior polymerization depth [22]. The reduction in polymerization shrinkage stress in bulk-fill composites leads to lower stress at the adhesive interface, thus maintaining bond strength over time [23].

Beyond the bond strength between restorative materials and dental hard tissues, the adhesion between repair and restorative materials is also critical for treatment outcomes [24,25]. While limited research has explored the impact of irrigation solutions on MTA [26,27], to our knowledge, no studies have specifically evaluated how final irrigation solutions influence the bond strength of MTA to bulk-fill composite. Thus, this study aimed to examine the effects of 17% EDTA, 2% CHX, 0.2% chitosan, 0.2% chitosan combined with 2% CHX, and 0.2% chitosan with AgNPs as final irrigants on the bond strength between MTA and bulk-fill composite. The null hypothesis was that these solutions would not impact the bond strength of MTA to the bulk-fill composite.

## 2. Materials and Methods

### 2.1. Sample Size Selection and Preparation

The sample size was calculated using G*Power V. 3.1.9.6 (Franz Faul, University of Kiel, Germany). Based on a 95% confidence level (1-α), a statistical power of 95% (1-β), and an effect size of f = 1.185, the minimum required sample size was determined to be 24, with 4 samples per group. To increase the robustness and reliability of the results, in line with previous study, 10 samples per group and 60 samples were included in the present study [28].

Sixty cylindrical cavities (5 mm diameter, 2 mm height) were created in a rigid plastic sheet using computer-aided design. MTA (Ortho-MTA; BioMTA, Seoul, Republic of Korea) was mixed as per the manufacturer’s instructions and placed into the cavities with endodontic plugs. Wet gauze was placed over the samples, and they were incubated at 37 °C with 100% humidity for one week to achieve full hardening. Afterward, the surfaces were polished under distilled water with minimal hand pressure. The samples were then randomly assigned to six groups (n = 10) according to the irrigation solution used: (1) EDTA: 17% EDTA (Sigma-Aldrich, St. Louis, MO, USA), (2) CHX: 2% CHX (Drogsan, Ankara, Turkey), (3) Chitosan: 0.2% chitosan solution, (4) Chitosan-CHX: 0.2% chitosan solution containing 2% CHX, (5) Chitosan-AgNP: 0.2% chitosan solution containing AgNPs, and (6) distilled water (Control).

### 2.2. Preparation of Chitosan-Based Solutions (Chitosan, Chitosan-CHX, Chitosan-AgNP)

**Figure 1:**
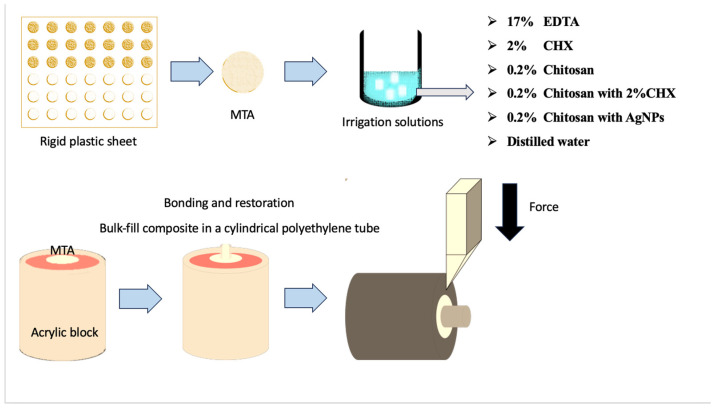
An illustration showing preparation of the specimens for shear bond strength test.

To prepare 100 mL of 0.2% chitosan solution, 0.2 g of medium-molecular-weight chitosan (Sigma Aldrich, St. Louis, MO, USA) was dissolved in a 1% lactic acid solution. The solution was then filtered using Whatman 42 filter paper (2.5 μm pore size) and stored at 4 °C until further use. The Chitosan-AgNP solution was synthesized via the chemical reduction method. Stock solutions of silver nitrate (AgNO_3_) and sodium borohydride (NaBH_4_) were prepared in distilled water [29]. For the preparation of 25 mL of Chitosan-AgNP dispersion, 1 mL of 0.01 M AgNO_3_ stock solution was combined with 23.5 mL of the 0.2% chitosan solution. The mixture was incubated and shaken at 50 °C for 60 min. Following this, 0.5 mL of freshly prepared NaBH_4_ solution was added to the reaction mixture. The solution was stirred continuously and left to react for an additional 90 min, during which a clear yellow-brown color developed, indicating the formation of Chitosan-AgNP. For Chitosan-CHX solution, 2 mL of 2% CHX was mixed with the chitosan solution to reach 100 mL. Samples were immersed in 5 mL of each irrigation solution for 5 min, rinsed with distilled water, and air-dried (Figure 1).

### 2.3. Restoration Procedure

**Figure 2:**
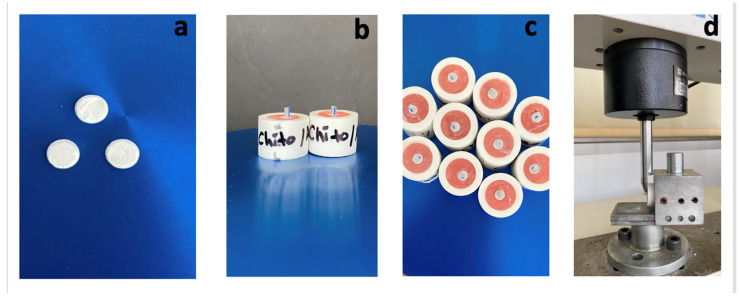
(**a**) MTA samples. (**b**) MTA samples embedded in cold acrylic and bulk fill composite in cylinder tubes. (**c**) Ready samples for bonding test. (**d**) Application of shear bond test on a sample.

All MTA specimens were exposed to irrigation solutions for 5 min, then embedded in cold acrylic resin in cylindrical acrylic blocks with the surfaces flush with the acryl (Figure 1). Next, a self-etch adhesive (Bond Force II; Tokuyama Dental, Tokyo, Japan) was applied to the MTA surfaces for 10 s, followed by gentle air drying and light curing for 10 s. A flowable bulk-fill composite (SDR, Dentsply, Konstanz, Germany) was then introduced into cylindrical polyethylene molds (2 mm × 2 mm) placed on the MTA surfaces and light-cured for 20 s (Figure 1 and Figure 2). The specimens were subsequently stored at 37 °C in 100% humidity for 24 h before conducting the shear bond strength test.

### 2.4. Shear Bond Strength Test and Failure Modes

Bond strength was measured using a universal testing machine (Shimadzu AG-1; Shimadzu Corp, Tokyo, Japan) at a constant rate of 1 mm/min parallel to the bond area until failure (N) (Figure 1 and Figure 2). Results were divided by surface area and recorded in megapascals (MPa). A stereo microscope (Leica MZ 16; Leica Microsystems GmbH, Wetzlar, Germany) at 20× magnification was used to classify failures: adhesive (no resin remnants between MTA and composite), cohesive (within MTA or composite), and mixed.

### 2.5. Microstructural and Elemental Analysis

Eighteen circular MTA specimens (5 mm diameter, 2 mm height) were created using computer-aided design in a rigid plastic sheet. The specimens were randomly divided into six groups (n = 3) and immersed in the final irrigation solutions, similarly to those used in the SBS test. After rinsing with distilled water, the samples were dried and gold-coated using an ion sputtering machine, and examined by scanning electron microscopy and energy-dispersive spectroscopy (SEM-EDX, Hitachi SU-5000, Hitachi, Tokyo, Japan). Surface properties and elemental composition were analyzed at 10,000× magnification and 10 kV.

### 2.6. Statistical Analysis

The data analysis was performed using IBM SPSS version 23. The Shapiro–Wilk test was used to assess data distribution normality. To evaluate measurement consistency, the intraclass correlation coefficient (ICC) was calculated. For data with normal distribution, one-way ANOVA was applied, with post hoc comparisons conducted using Tamhane’s T2 and Tukey tests. Results were presented as mean ± standard deviation and median (range), with a significance level set at α = 0.05.

## 3. Results

### 3.1. Shear Bond Strength Test

**Figure 3:**
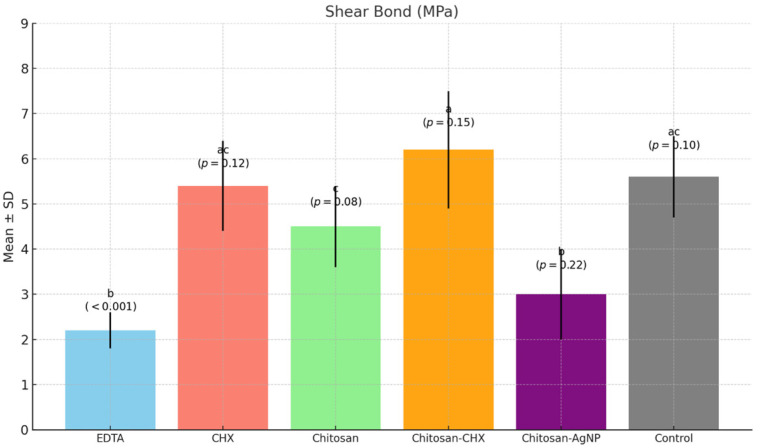
Bar chart showing shear bond strength values (MPa) (Mean ± SD) for each group, with statistical significance annotations (“a”, “b”, “c”) and corresponding *p*-values displayed.

The shear bond strength values of MTA samples after exposure to different final irrigation solutions are summarized in Table 1. A one-way ANOVA test revealed that there were statistically significant differences in SBS among the analyzed groups (*p* < 0.001) (Figure 3). Compared to the control group, the EDTA group showed the lowest SBS value (*p* < 0.001). Post hoc comparisons showed that there was no statistically significant difference between the EDTA and Chitosan-AgNP groups (*p* = 0.22). The highest SBS value was found in the Chitosan-CHX group, while there was no significant difference among the Chitosan-CHX, control, and CHX groups (*p* > 0.05). A significant difference was found among the chitosan-based groups (*p* < 0.001). The Chitosan-AgNP group had the lowest SBS value, while the Chitosan-CHX group had the highest SBS value (*p* < 0.001).

### 3.2. Fracture Types

**Figure 4:**
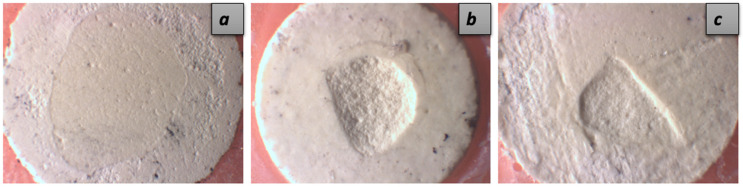
A representative image of the failure types: adhesive failure (**a**), cohesive failure in MTA (**b**), mixed failure (**c**).

The failure types rates of MTA after SBS test are shown in Table 2. In all groups, no cohesive fractures were observed in the bulk-fill restorative material itself. In general, the primary fracture type in the samples was cohesive in MTA, followed by mixed and adhesive fracture types. The highest frequency of mixed fractures was observed in the distilled water group, while the Chitosan-AgNP and EDTA groups showed the lowest frequency. The most adhesive fractures and the least mixed fracture types were observed in the Chitosan-AgNP group. Representative images of different failure types in each group are shown in Figure 4.

### 3.3. Microstructural Analysis of MTA

**Figure 5:**
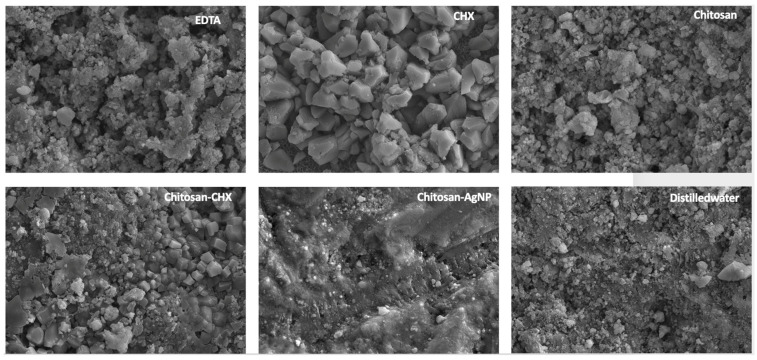
Scanning electron microscopy images of MTA specimens after exposure 17% EDTA, 2% CHX, 0.2% chitosan solution, 0.2% chitosan solution containing 2% CHX, 0.2% chitosan solution containing AgNPs, and distilled water (10,000× magnification and 10 kV).

Representative SEM images of MTA samples exposed to different final irrigation solutions are shown in Figure 5. The microstructure of MTA exposed to distilled water (control) appeared more amorphous. The microstructure of MTA treated with chitosan solution was similar to that of distilled water, but with more voids between the amorphous structures on its surface. A dominant cubic crystal structure was observed on the surface of MTA immersed in CHX solution, and a similar structure was noted on the surface of MTA exposed to the Chitosan-CHX solution. This solution combined features found in the microstructures induced by both chitosan and CHX. The most spongy structure, with a fragmented, void-rich appearance, was seen on the surface of MTA treated with EDTA. The group in which the crystal structure of MTA was disrupted, giving a flat appearance, was Chitosan-AgNP, where smaller, rounded structures were also seen between the crystals.

### 3.4. Elemental Analysis of MTA

**Figure 6:**
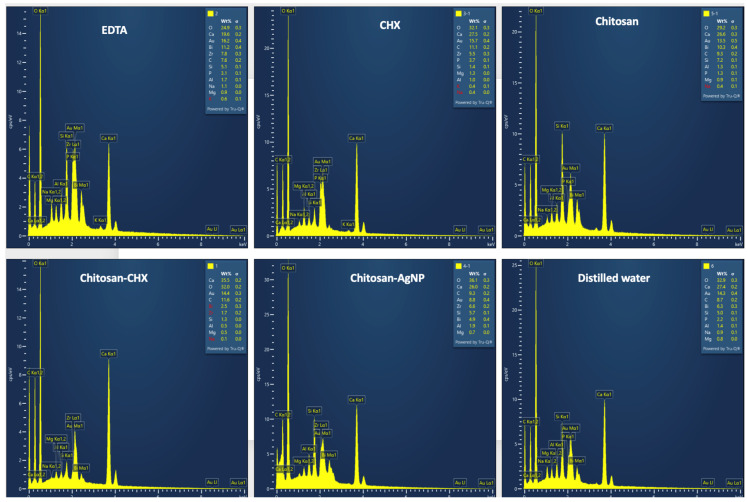
Representative energy-dispersive spectroscopy image of MTA treated with 17% EDTA, 2% CHX, 0.2% chitosan solution, 0.2% chitosan solution containing 2% CHX, 0.2% chitosan solution containing AgNPs and distilled water.

In the tested MTA cement, the Ca level showed the greatest reduction in the EDTA group compared to the other groups, while Ca ratios were similar across all other groups. Among the chitosan-based solutions, Ca ratios in MTA were consistent, with Chitosan-CHX showing the least decrease. The ratios of carbon, sodium, oxygen, aluminum, zirconium, and magnesium in the MTA specimens showed minimal variation across all experimental irrigation solutions. Representative energy-dispersive spectroscopy (EDS) images of the MTA specimens exposing with various final irrigation solutions are displayed in Figure 6.

## 4. Discussion

After the repair of apical plugs, perforations and root resorptions with tricalcium silicate cements, the chemo–mechanical preparation of the root canals usually needs to be continued. In this case, the repair materials were exposed to final irrigation solutions. These irrigants can compromise the structural stability of tricalcium silicate cements [7,26,27], potentially weakening their adhesion to restorative materials. This disruption may increase the risk of microleakage, allowing intraoral fluids and bacteria to infiltrate the root canal system [30]. Such leakage poses a significant risk for long-term endodontic treatment success, as persistent microbial ingress is a known factor in endodontic treatment failure [31,32]. Studies investigating the bonding between MTA and bulk fill composites are very limited in the literature (Table 3) [28,33,34,35]. In addition, there are no studies investigating the bonding of MTA with restorations after exposure to chitosan-based irrigation solutions and surface changes.

In this study, a clinical scenario was simulated in which root canals were irrigated with final irrigation solutions after the repair of perforations or resorption defects using MTA, followed by the placement of restorative material in the repaired area. For this purpose, prepared MTA samples were directly exposed to 17% EDTA, 2% CHX, 0.2% chitosan, 0.2% chitosan solution containing 2% CHX and 0.2% chitosan solution containing AgNPs solutions. Although there is no clear protocol in the literature regarding the optimal duration of use of irrigation solutions during chemomechanical procedures of the root canal after the placement and setting of MTA after perforation repair, different durations ranging from 1 min to 10 min have been applied in different studies [7,36,37,38,39,40]. Therefore, in this study, MTA samples were exposed to irrigation solutions for 5 min in parallel with several previous studies [37,39]. Then, the bulk-fill composite, which is preferred in post-endodontic restorations due to its low polymerization shrinkage, practicality of application, and homogeneous distribution in canal openings and access spaces [41], was applied on MTA and its bond strength was tested. In this study, as in many previous studies, the SBS test, which is a practical and safe method, was used to test the bond strength [35,42,43].

Significant differences in SBS values were found among the tested irrigation solutions, leading to the rejection of the null hypothesis. The SBS values between MTA and bulk fill composite after treatment with distilled water, which was the control group in the present study, were consistent with the previous studies investigating the SBS value between MTA and bulk fill composite without any surface treatment [35,44]. On the other hand, the lowest SBS value was observed in samples treated with 17% EDTA. Previous studies have reported that the microhardness of MTA may decrease, its structural integrity may change, and its bonding ability with resins may be adversely affected when exposed to chelating agents [28,45,46]. It has also been emphasized that its microstructure can become more porous and less crystalline [7,26]. In the current study, an SEM analysis revealed a more porous structure in MTA cements exposed with EDTA, likely due to the dissolution of interlocking crystalline components such as ettringite and portlandite [47,48]. Additionally, an elemental analysis of the MTA samples revealed a significant reduction in calcium ratios in the MTA specimens exposed to EDTA, compared to the other solutions. This observation showed that a strong chelating agent like EDTA disrupted the microstructure of MTA [7,49]. Such disruption may explain the lowest SBS value between MTA and bulk-fill composite by weakening the chemical adhesion with the restorative material [50].

In the literature, the bond strength of resin materials applied to MTA has been investigated in relation to various factors, including the type of adhesive system used, surface treatment of the MTA, and the time elapsed after MTA placement [24,25,51,52,53]. Acid etching of MTA prior to the application of restorative materials has been reported to enhance bonding by promoting micro-retention, which is critical for a durable bond, through the removal of irregular structures [52,53]. However, it is essential to balance the benefits of etching with the potential reduction in surface microhardness, as excessive etching may compromise the structural integrity of MTA [54,55]. In the current study, a 0.2% chitosan solution slightly reduced the SBS value between MTA and the bulk-fill composite compared to the control group, though this reduction was significantly lower than that caused by EDTA. This outcome may be explained by chitosan’s relatively mild chelating action compared to EDTA [56,57,58]. SEM images of MTA samples treated with chitosan displayed a less spongy and porous structure than those treated with EDTA, supporting this interpretation. In an elemental analysis, the calcium reduction was less pronounced in samples treated with chitosan and other chitosan-based solutions than in those treated with EDTA, suggesting that chitosan did not cause structural degradation during chelation. Chitosan is known to be effective only when dissolved in acid, and it has been shown that using different solvents can affect its physicochemical properties [59,60]. In this study, a 0.2% chitosan solution prepared with 1% lactic acid (pH 2–4) was preferred due to its low cytotoxicity, high biocompatibility, hydrophilicity, and significant antibacterial properties [59]. The slight decrease in SBS values in MTA exposed to chitosan compared to CHX, Chitosan-CHX, and distilled water may be attributed to the acidic pH and chelation property of this solution.

In the present study, the second-lowest SBS value after the EDTA group was observed in the Chitosan-AgNP group. This may be attributed to the structural degradation of MTA when exposed to a highly acidic environment [61,62]. The Chitosan-AgNP solution used in this study was prepared by adding AgNO_3_ to a 0.2% chitosan solution. Since AgNO_3_ was mildly acidic, the solution maintained an acidic pH. The detrimental effects of acidic environments on microhardness are well documented, and studies have shown that lower pH levels can lead to structural changes in MTA, increasing porosity and reducing its mechanical properties [4,55,61,62]. This reduction in microhardness may compromise the integrity of the bond formed with resin composites [63]. The addition of AgNP to chitosan has been reported to increase surface free energy, which is indicative of an increased surface area needed for optimal interaction [64]. Therefore, it is plausible that the addition of AgNP to chitosan increased the surface area of the solution, enhancing its penetration into the internal structure of MTA, thereby facilitating damage. The degraded crystalline structure and porous surface observed in SEM images of this group support this conclusion. All these adverse effects may have prevented the adhesion of the restorative material to the MTA surface. On the other hand, similar high SBS values were observed in samples treated with CHX, the Chitosan-CHX mixture solution, and distilled water, with the highest values found in the Chitosan-CHX group. Previous studies which investigated the effect of CHX solution on MTA reported that CHX reduced MTA’s microhardness less than EDTA and yielded microhardness values close to those of distilled water [27,65]. This indicated that CHX solution did not compromise the structural integrity of MTA cement in the same way that acidic or chelating agents did [27,61]. In this study, SBS values and calcium levels in the elemental analysis of the CHX group were not significantly affected, either positively or negatively, compared to the control group. However, the SEM analysis of the CHX group revealed cubic structures, which may be attributed to the slightly alkaline pH of CHX [10]. The exposure to an alkaline environment could lead to changes in the crystalline structure of MTA, potentially transforming it into a more distinct cubic prismatic form [66,67]. In the present study, the highest bond strength was observed with the 0.2% chitosan solution containing 2% CHX. This mixed solution combines the mildly acidic and weakly chelating properties of chitosan with the slightly alkaline pH of CHX [10,58]. As a result, this solution was expected to be less acidic and exhibited a reduced chelating effect compared to the 0.2% chitosan solution alone. These characteristics may have allowed the new mixed solution to subtly alter the surface morphology of MTA without compromising its structural integrity, thereby enhancing micromechanical retention. The SEM images of this group displayed features reflective of both the chitosan and CHX groups. The elemental analysis also revealed higher calcium levels compared to EDTA, confirming the weaker chelating effect [58].

Consistently with previous studies [25,34,68,69], no cohesive failure was observed within the bulk-fill composite in this study, while cohesive failures within MTA were more common, and adhesive failures were generally less frequent. This highlighted the effect of the weakening intrinsic strength of MTA on bond strength. Cohesive failure within the MTA reflected the inherent strength of the biomaterial and was independent of the bond strength between the restorative material and the tricalcium-silicate cement [25,70]. This may explain the predominance of cohesive fractures and minimal adhesive fractures observed in the EDTA and AgNP groups, regardless of the low SBS values. Consistently with previous studies, mixed fracture types were more frequently observed in the CHX, Chitosan-CHX, and distilled water groups, which showed higher SBS values [28,35].

The literature lacks an established threshold for acceptable bond strength between tricalcium silicate-based cements and restorative materials. Nevertheless, the findings of this study align with previous research [25,35,44]. Previous studies have shown that the choice of adhesive system and acid etching plays a critical role in the bond strength of MTA [24,71]. In the present study, the same restorative procedure was applied to all groups, with bonding performed without additional etching. Additionally, studies have shown that the timing of bonding procedures after MTA application is important [25,51,72]. It has been reported that bonding to freshly mixed MTA can compromise bond strength, whereas allowing MTA to set for a period before bonding significantly improves shear bond strength to resin composites [68]. Although the experimental procedure in this study was standardized to allow for the consistent setting times of MTA samples, the application time of irrigating solutions and the effect of body fluids on MTA’s setting time may vary under actual clinical conditions. Within the limitations of this study, clinical scenarios may not be simulated fully; however, such in vitro studies can provide valuable insights for future in vivo research. For example, recent advancements in microchip technology have significantly impacted research in the field of dental and endodontic biomaterials [73]. Modern organ-on-a-chip in vitro models, when combined with biomaterials such as bioactive glass, and calcium silicate-based cements, offer invaluable preclinical research tools. These models allow for more accurate simulations of the oral environment, including cellular interactions and tissue regeneration processes, which are critical for assessing the performance and biocompatibility of endodontic materials. The integration of these biomaterials into organ-on-a-chip platforms not only enhances the physiological relevance of the models but also provides a promising avenue for testing the efficacy of new dental treatments and interventions before clinical trials.

**Table 3:** Comparison of current studies.

Study	Biomaterial	Restoration Material	Methodology	Main Findings	Comments
Fatima et al. (2024) [24]	MTA, Biodentine, and CEM	-Bulk-fill composite	-Shear bond -Different restoration timing (15 min and 72 h) -Different adhesive systems (acid, no acid)	The highest bond strength of MTA was observed with two-step SE group at 15 min	Phosphoric acid application was found to increase the bond strength of MTA by increasing micro-retention.
Falakaloğlu et al. (2023) [28]	TheraCal PT TheraCal LC Biodentine NeoMTA 2 BioMTA	-Bulk-fill composite	-Shear bond	TheraCal PT showed the highest SBS	MTA samples showed low SBS values, but it was stated that the application of different surface treatments could increase SBS.
Palma et al. (2021) [25]	Biodentine, TotalFill BC Putty and PCM	-Bulk-fill composite	-Shear bond -Different restoration timing (immediate or 7 days delayed) -No acid	Biodentine immediate showed the highest SBS	It was found appropriate to perform restorative procedures immediately after biomaterial (3 or 12 min depending on the bioactive cement).
Ergül et al. (2024) [71]	ProRoot MTA, Medcem Pure Portland Cement, and Medcem MTA	-Conventional glass ionomer cement, resin-modified glass ionomer cement (RMGI), and bioactive restorative material	-Shear bond -With adhesive, without adhesive restoration	The highest shear bond strength value was noted in the Medcem MTA + ACTIVA bioactive	It has been suggested that the application of adhesives to calcium silicate-based biomaterials could effectively overcome the technical limitations.
Gürcan and Şişmanoğlu (2023) [52]	Biodentine, NeoMTA Plus, NeoPUTTY MTA, TheraCal LC, and Well-Root PT	Resin composite	-Shear bond -Surface treatment procedures before restoration	Aluminum oxide–treated TheraCal LC group exhibited the highest bond strength	The study suggested that surface treatments provide advantages in SBS in CSMs.
Uslu et al. (2024) [53]	MTA, Biodentine, Theracal LC, and Theracal PT	Bulk-fill composite	-Shear bond -Self-etch, acid etch, and laser etch before restoration	Theracal PT showed the highest bond strength. The acid-free-laser etch control group showed significantly lower bond strength compared to the acid etch and laser etch groups.	Acid or laser etching is recommended for effective adhesion.
Karaman et al. (2024) [35]	MTA and Theracal PT	Composite resin, compomer, and bulk-fill composite.	-Shear bond	Theracal PT showed significantly higher SBS than MTA	Theracal PT, with superior bond strength highlighted.
Çeliksöz and Irmak (2024) [44]	Biodentine RetroMTA	Glass hybrid material, resin composite and Theracal LC	-Shear bond -Different restoration time (3 min, 12 min, 24 h)	For both calcium silicate cements, the SBS obtained when the resin composite was applied at the end of all time periods was higher than that of the other materials.	For Biodentine; it was observed that there was no need to delay the restoration (24 h) in terms of bond strength and for RetroMTA; the bond strength with the restoration increased as the waiting time increased.
Candan et al. (2023) [42]	NeoMTA2, NeoPutty, and TheraCal PT	Three different resin composite materials	-Shear bond -Immediate-delayed restoration	TheraCal PT had the highest SBS values for both immediate and delayed restorations	The SBS values of fiber-reinforced and nanohybrid composite materials for immediate and subsequent restoration of calcium silicate-based materials were found to be similar.
Naiboğlu et al. (2023) [54]	Biodentine, MTA, Angelus MTA, ProRoot MTA and MTA Repair HP	Composite resin	-Shear bond -Self-etch, etch and rise	MTA-P demonstrated significantly higher SBS than all groups.	MTA-P was considered a more suitable pulp capping material due to its superior SBS compared to BD and MTA-A.
This study	Ortho MTA	Bulk-fill composite	-Shear bond -Different irrigation solution (EDTA, CHX, chitosan-based solution) treated before restoration	Treatment with chitosan solution supplemented with CHX showed the highest SBS.	It has been emphasized that chitosan and chitosan with CHX irrigation solutions can be an alternative to EDTA in endodontic procedures using MTA.

## 5. Conclusions

The aim of this study was to evaluate the bond strength of MTA to a bulk-fill composite when 17% EDTA, 2% CHX, 0.2% chitosan, 2% CHX with 0.2% chitosan, and AgNP with 0.2% chitosan were used as final irrigation solutions after an endodontic procedure requiring MTA application. The null hypothesis tested was that these solutions would not affect the binding of MTA to the bulk-fill composite differently. However, this study revealed that different irrigation solutions may differentially affect the bond strength of MTA and also emphasized the importance of careful management of chitosan-based irrigation solutions to maintain the integrity of MTA. The findings reiterate the importance of selecting the preferred irrigation solution after repair of iatrogenic perforations or pathological root resorption with MTA and suggest that the use of 0.2% chitosan solution alone or a milder chelating agent in combination with CHX may increase the bond strength for subsequent restorations and potentially improve the durability and success of MTA-based treatments.

Additionally, the implications of these findings extend beyond endodontics into the realm of dental prosthetics. The bond strength between MTA and bulk-fill composites is critical not only for the immediate success of the restoration but also for the longevity and functionality of subsequent prosthetic treatment, such as endocrowns, crowns, bridges, and other restorations. Improving the bond strength between MTA and restorative materials could enhance the durability and esthetics of prosthetic restorations, particularly in cases requiring complex endodontic interventions followed by prosthetic rehabilitation. Thus, the selection of appropriate irrigation solutions post-endodontic procedures could play a crucial role in the long-term success of both endodontic treatments and the dental prostheses.

In conclusion, this study contributes to the understanding of how irrigation protocols can affect the performance of MTA in clinical scenarios, particularly when followed by restorative procedures involving composite materials. Further research into the long-term implications of these findings is warranted to optimize treatment protocols and ensure the success of MTA-based interventions in both endodontics and prosthodontics.

## Figures and Tables

**Table 1:** Shear bond strength values (MPa) of the MTA specimens to bulk-fill composite after treatment with different final irrigation solutions.

Groups (*n* = 10)	Mean ± SD	Median (Min.–Max.)	Test Statistic	*p* *
EDTA	2.2 ± 0.4 ^b^	2.1 (1.7–3.1)	27.659	<0.001
CHX	5.4 ± 1 ^ac^	5.5 (4–6.8)		
Chitosan	4.5 ± 0.9 ^c^	4.5 (3.1–5.7)		
Chitosan-CHX	6.2 ± 1.3 ^a^	5.9 (4.8–9)		
Chitosan-AgNP	3 ± 1 ^b^	2.7 (1.8–4.8)		
Control (Distilled Water)	5.6 ± 0.9 ^ac^	5.5 (4.5–7.4)		

* One-way analysis of variance (ANOVA) was performed. Groups that share the same letter (a–c) indicate no statistically significant difference between them.

**Table 2:** Failure types of the MTA Specimens.

Groups (*n* = 10)	Adhesive	Cohesive in MTA	Cohesive in Bulk-Fill	Mixed Failure
EDTA	1	7	-	2
CHX	-	3	-	7
Chitosan	2	4	-	4
Chitosan-CHX	-	2	-	8
Chitosan-AgNP	1	8	-	1
Control (Distilled Water)	-	3	-	7

## Data Availability

All essential data are presented in the manuscript. The datasets used and/or analyzed during the current study are available from the corresponding author on reasonable request.
